# RNA modifying enzymes shape tRNA biogenesis and function

**DOI:** 10.1016/j.jbc.2024.107488

**Published:** 2024-06-20

**Authors:** Sarah K. Schultz, Ute Kothe

**Affiliations:** 1 Department of Chemistry, University of Manitoba, Winnipeg, Manitoba, Canada; 2 Alberta RNA Research and Training Institute (ARRTI), Department of Chemistry and Biochemistry, University of Lethbridge, Lethbridge, Alberta, Canada

**Keywords:** RNA, transfer RNA (tRNA), RNA modification, RNA methylation, RNA processing, RNA binding protein, precursor tRNA (pre-tRNA), aminoacyl tRNA synthetase, ribosome, protein synthesis, RNA folding, RNA structure

## Abstract

Transfer RNAs (tRNAs) are the most highly modified cellular RNAs, both with respect to the proportion of nucleotides that are modified within the tRNA sequence and with respect to the extraordinary diversity in tRNA modification chemistry. However, the functions of many different tRNA modifications are only beginning to emerge. tRNAs have two general clusters of modifications. The first cluster is within the anticodon stem-loop including several modifications essential for protein translation. The second cluster of modifications is within the tRNA elbow, and roles for these modifications are less clear. In general, tRNA elbow modifications are typically not essential for cell growth, but nonetheless several tRNA elbow modifications have been highly conserved throughout all domains of life. In addition to forming modifications, many tRNA modifying enzymes have been demonstrated or hypothesized to also play an important role in folding tRNA acting as tRNA chaperones. In this review, we summarize the known functions of tRNA modifying enzymes throughout the lifecycle of a tRNA molecule, from transcription to degradation. Thereby, we describe how tRNA modification and folding by tRNA modifying enzymes enhance tRNA maturation, tRNA aminoacylation, and tRNA function during protein synthesis, ultimately impacting cellular phenotypes and disease.

Transfer RNAs (tRNAs) are essential molecules that act as physical adapters between the genetic code and amino acids in every cell. Almost all tRNAs feature a cloverleaf secondary structure that folds into an L-shaped tertiary structure (Fig. 1*A*). Decades of ongoing research has established that all tRNAs contain modifications, which play a variety of roles in tRNA structure and function, and which are introduced by a diverse set of dedicated tRNA modifying enzymes (1, 2, 3, 4). Underlying the abundance and energy investment of tRNA modification within cells, genes encoding tRNA modifying enzymes can account for 1% of the protein coding genes in an organism. For example, the model organism *Escherichia coli* has ∼4300 protein coding genes, of which 59 encode enzymes involved in the formation of 28 tRNA modifications (Fig. 1*B*). Highlighting the importance of tRNA modification, mutations within tRNA sequences that prevent modification or within the genes encoding tRNA modifying enzymes have been implicated in a growing number of human diseases (5, 6, 7).

**Figure 1:**
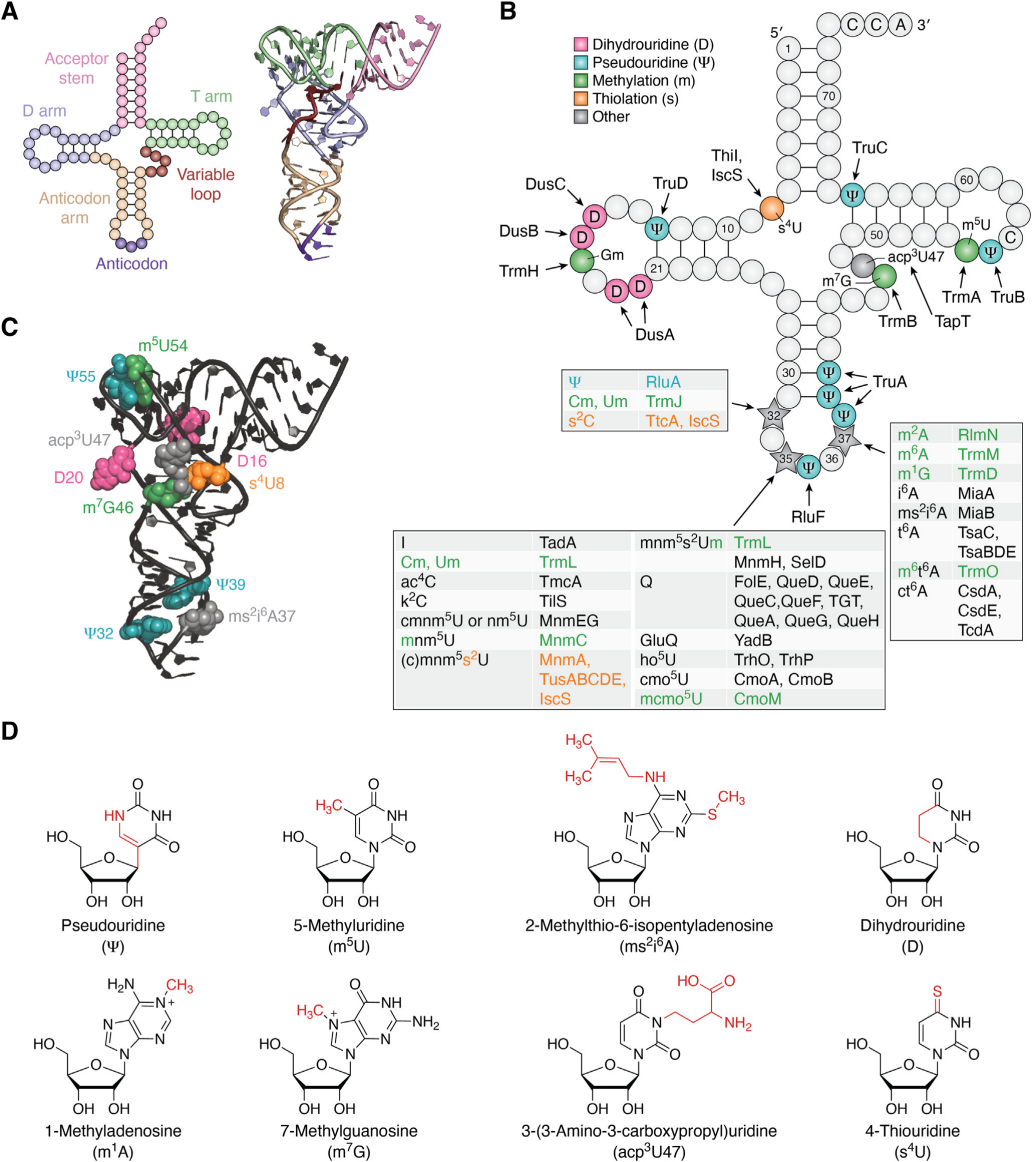
**tRNA structure and modification.***A*, cloverleaf secondary structure (*left*) and L-shaped tertiary structure (*right*) typical of the majority of tRNAs. *B*, locations and identities of all modifications in *Escherichia coli* tRNAs. Common modifications are denoted by color, as indicated in the legend, which also indicates the abbreviations for these common modifications. Modifications are systematically abbreviated with letters preceding the nucleotide indicating a base modification. Superscript numbers indicate the position of the nucleotide where the modification is found. Letters after the nucleotide indicate modification to the ribose sugar. More explanation for the abbreviation of tRNA modifications is excellently summarized in (1). Multiple different modifications can occur at positions 32, 34, and 37 in different tRNAs, as shown in tables. Abbreviations not included in the legend but present in the table are as follows: acp–aminocarboxypropyl, I–inosine, ac–acetyl, k–lysidine, (c)mnm–(carboxy)methylaminomethyl, Se–selenium, Q–queuosine, ho–hydroxy, cmo–carboxymethoxy, mcmo–methoxycarbonylmethoxy, i–isopentyl, (c)t–(cyclic)threonylcarbamoyl. *C*, locations of modifications within the *E. coli* tRNA^Phe^ structure (82) as a typical tRNA to demonstrate clusters of modifications in the tRNA elbow and in the tRNA anticodon stem-loop. Modifications are colored as per the legend in panel *B*. *D*, examples of some of the modifications found within tRNAs. Atoms that constitute each modification are indicated in *red*.

Within the L-shaped three-dimensional tRNA structure, two clusters of tRNA modifications are apparent (Fig. 1*C*) (1, 8). The first cluster of tRNA modifications is found within the tRNA anticodon stem-loop. In this region, modifications often play direct functional roles during protein translation (9, 10). For this reason, several modifications found within this region are essential for cell viability. The second cluster of tRNA modifications is evident in the elbow of the L-shaped tertiary structure, which is also referred to as the tRNA body (8). In general, modifications within this region fine-tune tRNA structure and stability (1).

Modifications to tRNA are often simple reactions, such as isomerization of the uracil base to form pseudouridine, reduction of uridine to form dihydrouridine, methylation at various atoms of the nucleobase or ribose sugar, or exchange of oxygen for sulfur during thiolation; however, some reactions are more complex and involve more than one enzyme, forming bulky hypermodifications (Fig. 1*D*). Most often, tRNA hypermodifications are found within the tRNA anticodon loop, especially at position 34, which is the wobble position that base pairs to the nucleotide at the third position of an mRNA codon during translation, and at the conserved purine at position 37, which is adjacent to the anticodon. Certain modifications to the tRNA elbow are found in many tRNAs across all domains of life, suggesting a conserved function. These include dihydrouridine (D) at various positions within the D arm, 5-methyluridine (m^5^U, also known as ribothymidine, rT) 54, pseudouridine (Ψ) 55, 1-methyladenosine (m^1^A) 58, 2ʹ-O-methylguanosine (Gm) 18, and 7-methylguanosine (m^7^G) 46. In contrast, modifications within the tRNA anticodon loop are often found in only a handful of tRNAs and display more variation between different organisms, suggesting possible roles for certain modifications in environmental adaptation. In the model organism *E. coli*, all tRNA modifications are thought to be mapped, and with the identification of the TapT enzyme in 2019, all enzymes responsible for *E. coli* modifications have been identified (11, 12, 13). In contrast, complete mapping of tRNA modifications in most organisms has yet to be accomplished and novel modifications and modifying enzymes remain to be discovered.

The majority of tRNA modifying enzymes have been found to be nonessential for cells grown in ideal laboratory conditions (14); however, many cellular phenotypes have been identified for tRNA modification enzyme knockout (KO) strains in stress conditions, suggesting an importance for these enzymes in maximizing cellular fitness (Table 1). Moreover, deletion of two or more modification enzymes oftentimes results in more severe growth defects, emphasizing the likely redundant nature of tRNA modifications and modifying enzymes (15). In addition to the catalytic role of modifying tRNAs, certain tRNA modification enzymes may also play an important role in folding tRNAs, acting as tRNA chaperones (16, 17, 18). Thus, tRNA modifying enzymes not only affect the modification status of tRNAs, but also tRNA folding. tRNA interacts with a variety of factors during its lifecycle (Figs. 2 and 3) and tRNA modification status and folding affect several of these interactions. In this review, we summarize the precise roles of tRNA modifying enzymes and tRNA modifications with respect to the tRNA lifecycle. In particular, we focus on how modifications and the responsible enzymes collaborate to affect tRNA maturation and function during translation.

**Table 1:** Stress-related growth phenotypes for tRNA modification deletion strains

tRNA modification	tRNA modifying enzyme	Organism	Phenotype	Ref.
Ψ55	TruB	*Escherichia coli*	Reduced fitness in coculture competition	(17, 86, 174)
			Impaired recovery from heat shock	
Ψ55	TruB	*T. thermophilus*	Slow growth at 50 °C (cold for thermophilic model organism)	(175)
m^5^U54	TrmA	*E. coli*	Reduced fitness in coculture competition	(16, 176)
			Increased growth in the presence of hygromycin	
m^5^U54	Trm2	*Saccharomyces cerevisiae*	Increased growth in the presence of hygromycin	(176)
m^7^G46	TrmB	*E. coli, Pseudomonas aeruginosa, Acinetobacter baumannii, Colletotrichum lagenarium*	Slow growth in presence of H_2_O_2_	(177, 178, 179, 180)
m^7^G46	TrmB	*T. thermophilus*	Slow growth at 80 °C	(181)
m^7^G46	Trm8/Trm82	*S. cerevisiae*	Slow growth at 38 °C in minimal media with 2% glycerol; augmented temperature sensitive growth in absence of *trm4*, *pus7*, or *dus3* genes	(15, 182)
Ψ38/39	Pus3	*S. cerevisiae*	Slow growth at 37 °C	(183)
t^6^A37	Tsc2/Tsc3	*S. cerevisiae*	Several phenotypes, including slow growth at 37 °C and slow growth in presence of TOR inhibitors	(117)
ncm^5^/mcm^5^34	Elongator complex	*S. cerevisiae* and *Schizosaccharomyces pombe*	Several phenotypes, including slow growth and temperature sensitivity	(184)
D16	DusC	*E. coli*	Slow growth at cold temperature (24 °C), exacerbated by loss of TmcA (forms ac^4^C)	(185)
m^5^C34 and m^7^G46	Nsun2 and METTL1	*Homo sapiens* (HeLa cells)	Increased sensitivity to 5-fluorouracil	(186)
m^1^A9	Trm10	*S. cerevisiae*	Increased sensitivity to 5-fluorouracil, particularly at 38 °C (along with additional tRNA modifying enzymes)	(187)

**Figure 2:**
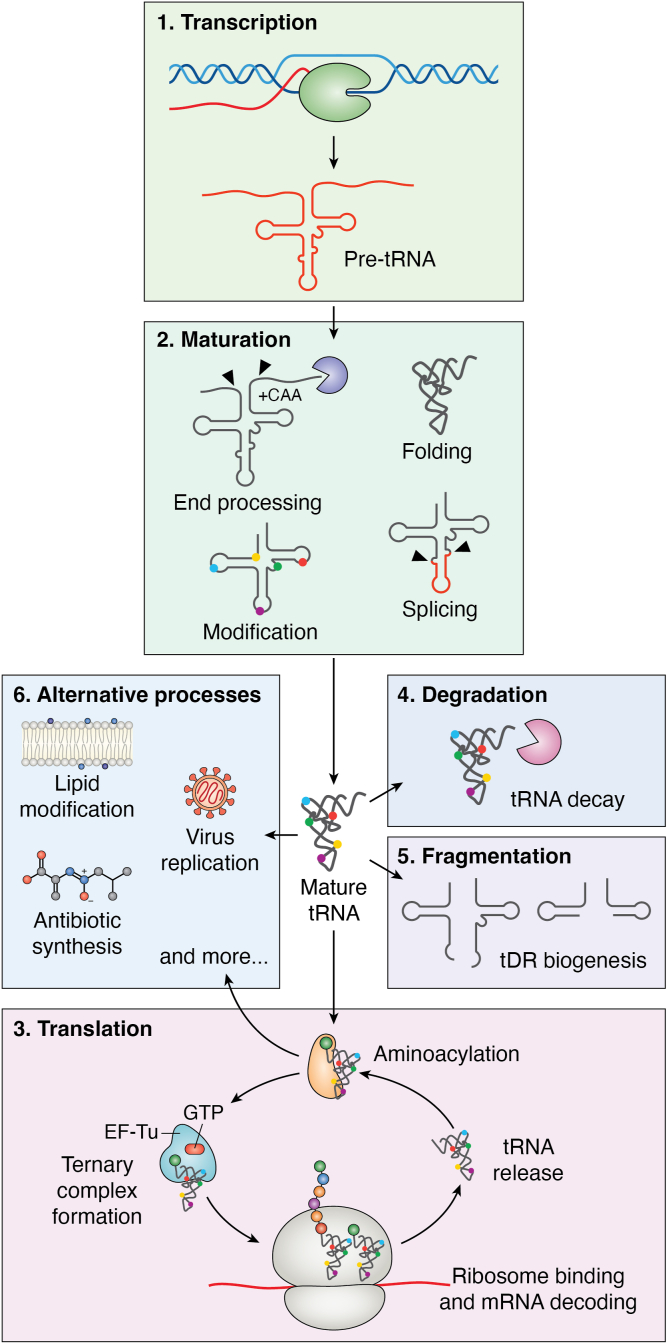
**General lifecycle of a tRNA.** Following transcription of pre-tRNA by RNA polymerase (1), tRNA undergoes several maturation steps (2) including 5ʹ and 3ʹ end processing, intron splicing, modification of many nucleotides, and RNA folding, giving rise to a mature tRNA. Mature tRNA is then aminoacylated to form aminoacyl-tRNA (aa-tRNA), which binds EF-Tu•GTP, forming the ternary complex for delivery to the ribosome (3). Various ribonucleases target tRNA to degrade aberrantly matured tRNA (4) or to cleave tRNA into functional tRNA-derived small RNAs (tDR) (5). Finally, tRNAs also participate in a variety of nontranslation alternative processes (6).

**Figure 3:**
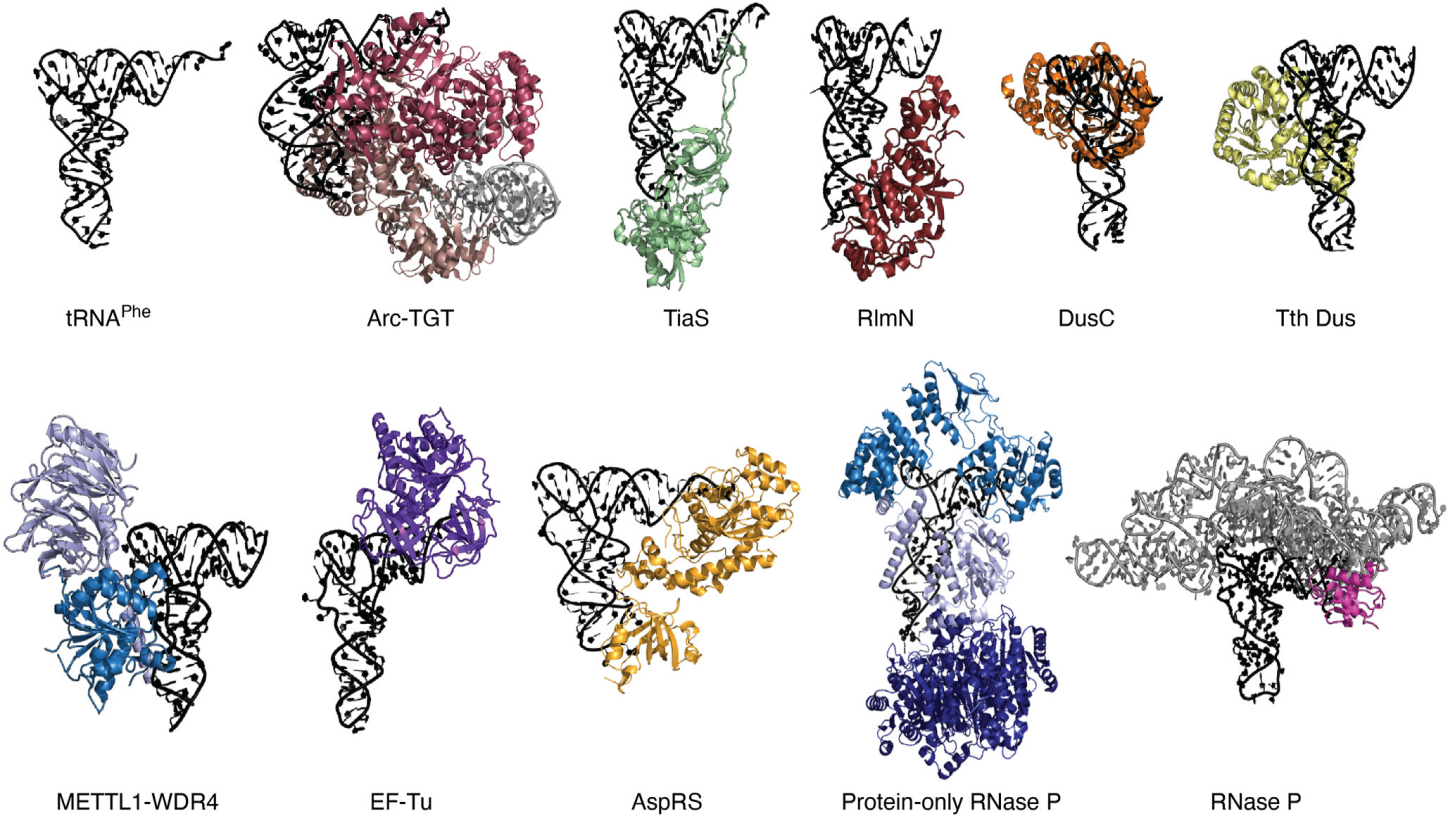
**Examples of tRNA-protein complex structures illustrating the various interactions of tRNAs throughout its lifecycle.** In all cases, tRNA is colored *black* and is in roughly the same orientation to demonstrate how different proteins interact with various surfaces of the tRNA. From *left* to *right* on the top row: yeast unbound tRNA (PDB: 4TNA) (237); *Pyrococcus horikoshii* ArcTGT homodimer colored in shades of *red* bound to two tRNA^Val^ molecules to modify G15 with tRNA adopting the lambda form. The second tRNA molecule is shown in *gray* (1J2B) (89); *Archaeoglobus fulgidus* TiaS bound to tRNA^Ile2^ for modifying C34 (PDB: 3AMT) (238); *Escherichia coli* RlmN bound to tRNA^Glu^ for modification of A37 (PDB: 5HR7) (239); *E. coli* DusC bound to tRNA^Trp^ to modify U16 (PDB ID: 4YCP) (240) and *Thermus thermophilus* Dus bound to tRNA^Phe^ to modify U20 (PDB: 3B0V) (241) displaying how different dihydrouridine synthases target different tRNA residues. The second line contains the METTL1 (*dark blue*) and WDR4 (*light blue*) heterodimer bound to tRNA to modify G46 (PDB: 8EG0) (242); *Thermus aquaticus* EF-Tu bound to tRNA^Cys^ (PDB: 1B23) (243); yeast AspRS bound to tRNA (PDB: 1ASZ) (244); human mitochondrial protein only RNase P in complex with pre-tRNA^Tyr^. RNase P is colored in *dark blue*, TRMT10C is colored in *light blue*, and MRPP3 is colored in *indigo* (PDB: 7ONU) (43); and *Thermotoga maritima* RNase P in complex with tRNA^Phe^ (PDB: 3Q1Q) (245). The protein component is colored in *pink* and the RNA in *gray*. PDB, Protein Data Bank.

## tRNA maturation overview

tRNAs are transcribed as precursor (pre)-tRNAs, which undergo a multienzyme maturation process prior to fulfilling diverse functions (Fig. 2). These maturation events are not strictly temporally defined, and the order can vary based on both organism and tRNA identity. In eukaryotes, the subcellular localization of tRNA processing components largely dictates the tRNA processing order. For example, in yeast many tRNAs are transcribed in the nucleolus whereas others may be transcribed in the nucleoplasm (19). In other eukaryotes, the details regarding the location of tRNA transcription and maturation remain unexplored. Nonetheless, tRNAs subsequently encounter different processing enzymes within the nucleus and cytoplasm, followed by potential retrograde import to the nucleus and reexport back to the cytoplasm (2, 20). Following transcription, the 5ʹ leader and 3ʹ trailer sequences are removed from the pre-tRNA by various endonucleases and exonucleases (21, 22, 23, 24). With the exception of several bacteria, the tRNA genes from many organisms lack the 3ʹ-CCA tail necessary for aminoacylation (25). As such, this sequence must be posttranscriptionally added by the tRNA nucleotidyltransferase (26). Certain tRNAs contain an intron which is usually located within the tRNA anticodon stem-loop (2, 27, 28). Eukaryotic and archaeal tRNA introns require several enzymes to excise the intron and repair the tRNA; however, the details of these reactions differ between organisms, and the definitive cellular location of tRNA splicing remains to be determined for several cell types (29, 30, 31). For example, whereas it has been postulated that tRNA splicing occurs in the nucleus in *Xenopus* oocytes and human cells (32, 33), more recent evidence using endogenous factors suggests splicing of pre-tRNA in mammalian cells occurs in the cytoplasm (34). Moreover, the tRNA splicing machinery is localized to the cytoplasmic surface of mitochondria in yeast (35). Bacterial tRNAs rarely contain introns, but when present they are self-spliced without the need for proteins (36).

Ribose and base modifications in tRNA can occur at all steps of tRNA maturation. Again, the intracellular localization of the responsible modifying enzyme plays a role in whether a modification will occur during the early or late steps of tRNA maturation, and in many cases, it may be that a tRNA is modified by a given enzyme at the first available opportunity within the cell, rather than in a strict order (37). In the two sections below, we will note instances of collaboration between tRNA modifying enzymes and crosstalk of modifications themselves with other enzymes involved in tRNA maturation, with a focus on how early tRNA modifying enzymes affect later acting modification enzymes.

## Cleavages of pre-tRNA: interplay between modifying enzymes and tRNA processing

Decades ago, it was first shown that many tRNA modifications occur prior to tRNA end processing and tRNA intron splicing in *Xenopus* oocytes (32, 38, 39). As such, it is unsurprising that crosstalk exists between tRNA modifying enzymes and other enzymes involved in tRNA maturation. Even a link between tRNA transcription and modification has been identified as changes in RNA polymerase III activity correlate with the activity of the tRNA dimethyltransferase Trm1 (40). In yeast, the 5ʹ end is almost always removed prior to the 3ʹ trailer, but in *E. coli*, an initial cleavage event at the 3ʹ end often precedes 5ʹ processing by RNase P (23, 24, 27, 28). Although RNase P is usually a ribonucleoprotein, certain organisms and organelles instead rely on an endonuclease comprised solely of proteins and thus called protein only RNase P (41) (Fig. 3). Interestingly metazoan mitochondrial protein only RNase P is a multienzyme complex composed of TRMT10C (also known as MRPP1), MRPP2, and PRORP (also known as MRPP3), wherein TRMT10C is a tRNA methyltransferase responsible for *N*^*1*^-methylation of purines at position 9 (42). Within this RNase P complex, TRMT10C forms contacts with all four tRNA arms, in addition to MRPP2 and PRORP (Fig. 3) (43). Presence of TRMT10C is necessary for efficient cleavage by RNase P by properly orientating PRORP and pre-tRNA, consequently enhancing the rate of nuclease activity (44). For certain pre-tRNA substrates, prior binding of SAM by TRMT10C may enhance pre-tRNA binding and cleavage (45); however, the methyltransferase activity of TRMT10C does not contribute to tRNA binding or activity of protein only RNase P (46). TRMT10C can methylate independently of PRORP, but for certain pre-tRNAs the presence of PRORP may increase the rate of methylation (45). Although the functional interdependence (if any) between the two tRNA maturation activities of TRMT10C remains unclear (44, 46), it has been hypothesized that TRMT10C may function as a platform for coordinating tRNA maturation events, including methylation, 5ʹ cleavage, in addition to 3ʹ processing by RNase Z and the CCA adding enzyme (47). In a similar manner, the plant single-subunit protein only RNase P has been found to interact with the dimethyltransferases TRM1A and TRM1B and these methyltransferases seem to additionally interact with RNase Z (48). In contrast to the role of TRMT10C in human mitochondrial RNase P, TRM1A/TRM1B interacts with plant RNase P in an indirect, tRNA-dependent manner, which nonetheless highlights coordination between tRNA modifying enzymes and tRNA processing enzymes as a theme during tRNA maturation.

Intron splicing occurs independently of tRNA end processing and modifications (27). Although several tRNA modification enzymes are insensitive to the presence or absence of tRNA introns, others absolutely rely on intron presence (or absence) for catalysis, as summarized in (49). For example, pseudouridylation of U35 in tRNA^Tyr^ by Pus1 within various eukaryotes absolutely requires intron presence but does not require the intron to be of a certain size or sequence (50, 51, 52), and C5-methylation of yeast tRNA^Phe^ at cytosine 40 requires an intron, but is not sensitive to the presence of the D or T arms (53). As far as currently known, the activity of the tRNA splicing machinery is unaffected by tRNA modifying enzymes.

## Early tRNA modifications affect the activity of later acting tRNA modifying enzymes

Approximately 10% of all nucleosides within tRNA are posttranscriptionally modified by a large set of dedicated tRNA modifying enzymes. Each tRNA contains its own unique set of modifications, but certain modifications, including m^5^U54, Ψ55, m^7^G46, and D at several positions, are found in many tRNAs (8). As suggested above, tRNA modifying enzymes may introduce their modification at any time during tRNA maturation (32, 38, 39), although a growing number of studies suggest that the modification process is at least somewhat ordered, with certain modifications appearing prior to tRNA end processing and splicing and others forming only after the pre-tRNA is processed. However, it is unlikely that tRNA modification follows a strict sequential order, as many tRNA modifying enzymes can effectively modify *in vitro* transcribed (*i.e.* unmodified) tRNA. Several instances of tRNA modifications positively and negatively affecting the activity of enzymes introducing other modifications have been uncovered and have been referred to as “modification crosstalk,” “modification circuits,” or “modification networks” (54, 55, 56, 57, 58). Determining the relative temporal order of tRNA modification is a challenging task because tRNA modifications are introduced quickly and because there are several technical difficulties. For example, it is necessary to overcome challenges in purifying specific tRNA isoacceptors, obstacles of quantitatively distinguishing sites of modification (*e.g.* distinguishing sites of several pseudouridines), and the need for sophisticated expertise and equipment to monitor many chemically distinct nucleotides in one experiment. Most often, modification crosstalk has been uncovered by extracting total tRNA or a specific tRNA isoacceptor from deletion strains lacking one (or more) tRNA modification enzyme. This tRNA is then monitored for changes in the steady-state abundance of other modifications, typically by using either high-performance LC/MS or various next-generation sequencing techniques (59). In other cases, *in vitro* studies have been used to determine how the presence/absence of modifications affects tRNA binding and modification by modifying enzymes; this approach can determine the mechanisms underlying tRNA modification crosstalk. Known instances of tRNA modifications positively and negatively affecting the activities of tRNA modifying enzymes that have been examined using these and similar assays are summarized in Table 2. As several reviews have explained modification circuits with an emphasis on tRNA anticodon stem-loop modifications (54, 55, 56, 57, 58), we summarize here recent techniques that have advanced our knowledge regarding the temporal placement of tRNA modifications with a focus on particular modifications within the tRNA variable and T loops: m^7^G46, m^5^U54, Ψ55, and m^1^A58.

**Table 2:** Interactions between tRNA modifications and tRNA modifying enzymes

Initial modification (enzyme)	Affected modification (enzyme)	Interaction+/−a	Organisms	Evidence	Ref.
m^1^A58 (TRM6/TRM61)	m^5^U54 (TRMT2A)	+	*H. sapiens* (HEK293FT)	LC/MS analysis of *TRM6* mutant cell lines	(66)
m^1^A58 (TrmI)	s^2^m^5^U54 (TtuABC, IscS)	+	*T. thermophilus*	LC/MS and enzymatic analysis of KO strain	(188)
Ψ55 (Pus4)	m^5^U54 (Trm2),	+	*S. cerevisiae*	LC/MS analysis of KO strain	(60)
	m^1^A58 (Trm6/Trm61)				
Ψ55 (TruB)	Gm18 (TrmH) m^5^s^2^U54 (TtuABC, IscS),	−	*T. thermophilus*	LC/MS and enzymatic analysis of KO strain	(175)
	m^1^A58 (TrmI)				
Ψ55 (TruB)	m^5^U54 (TrmA)	+	*E. coli*	Increased affinity of TrmA to Ψ55 tRNA *in vitro*	(74)
m^5^U54 (Trm2)	m^1^A58 (Trm6/Trm61)	+	*S. cerevisiae*	LC/MS analysis of KO strain	(60)
m^5^U54 (TrmA), s^4^U8 (ThiI), m^7^G46 (TrmB)	Ψ55 (TruB)	−	*E. coli*	Decreased affinity and activity of TruB for singly modified tRNA *in vitro*	(74)
m^7^G46 (TrmB)	acp^3^U47 (TapT)	+	*E. coli*	LC/MS and primer extension analysis of KO strain	(13)
m^7^G46 (TrmB)	Gm18 (TrmH) m^1^G37 (TrmD)	+	*T. thermophilus*	LC/MS and enzymatic assays of KO strain	(181)
i^6^A37 (MiaA)	(C/U)m34 (TrmL)	+	*E. coli*	LC/MS of bulk and specific tRNAs from *ΔmiaA* strains; *in vitro* activity assays	(189, 190)
t^6^A37 (TsaBDE)	L34 (TilS)	+	*E. coli*	Increased TilS activity *in vitro*	(191)
t^6^A37 (KEOPS)	mcm^5^s^2^U34 (Elongator)	-	*S. cerevisiae*	HPLC analysis of KO strains	(117)
i^6^A37 (MOD5) or t^6^A37 (KEOPS)	m^3^C32 (Trm140)	+	*S. cerevisiae*	tRNA-HySeq and LC/MS analysis of KO strains; tRNA pulldowns; *in vitro* methylation assays	(109, 192)
			*S. pombe*		
t^6^A37 (Sua5/KEOPS)	m^3^C32 (METTL2A)	+	*H. sapiens* (cell line HEK293T)	*In vitro* methylations assays	(108)
m^1^G37 (TrmD)	mcmo^5^U34 (CmoM)	+	*E. coli*	Faster *in vitro* methylation	(73)
Q34 (Tgt)	m^5^C38 (Dnmt2)	+	*S. pombe*	*In vivo* bisulfite sequencing of KO strains	(142, 193)
			*D. discoideum*		
			*H. sapiens* (HeLa and HCT116 cell lines)		
Cm32 and Gm34 (Trm7 and Trm732 or Trm734)	yW37 (yeast, Tyw1-4) or o2yW37 (humans, unknown)	+	*S. cerevisiae*	LC/MS and biochemical characterization of tRNA from KO strains	(194, 195, 196, 197)
			*S. pombe*		
			*Mus musculus*		
			*H. sapiens*		
m^3^C32 (Trm140)	m^3^U32 (ADAT2/3)	+	*T. brucei*	*In vitro* modification assays show U32 methylation must occur prior to C-to-U editing and Trm140 must be present for editing reaction to occur	(198)
Deamination of m^3^C32 to m^3^U32	I34 (ADAT2/3)	+	*T. brucei*	More efficient inosine formation *in vitro*	(199)
One or more of the full modification set	D20 (Dus2)	+	*S. cerevisiae*	Tighter binding of modified tRNA and faster oxidation of Dus2	(200)

Abbreviation: KO, knockout.

a + indicates modification whose presence is stimulated by the enzyme identified in the first column. - indicates a modification whose presence is repressed by the enzyme identified in the first column.

Several studies have identified that T arm modifications, in particular m^5^U54 and Ψ55, generally tend to be among the earliest introduced during tRNA maturation. Recently, a time-resolved NMR assay was developed to follow the modification of a stable-isotope labeled *in vitro* transcribed tRNA^Phe^ molecule within yeast cell extract (60). This experiment identified that Ψ55 is introduced early and quickly into tRNA, followed by m^5^U54 (over a long period of time) and m^7^G46 (over a short period of time), followed by m^2^G10, m^5^C49, and D16. Finally, m^1^A58 was the last modification to be detected within tRNA^Phe^ (m^2^_2_G26 and m^5^C40 were not observed within the incubation timeframe). Although this experiment is able to detect many modifications at one time in a cell-like environment, disadvantages include loss of cellular compartmentalization, meaning tRNA may encounter cytoplasmic enzymes sooner than it would within the cell. An interesting finding of this study is the apparently late introduction of the m^1^A58 modification. Considering the enzymes responsible for m^1^A58 (Trm6/Trm61) are localized to the nucleus (61), it would be surprising for m^1^A58 to be one of the last modifications to be introduced. Moreover, methylation of A58 in initiator tRNA is well-known to protect initiator tRNA^Met^ from nuclear decay (61, 62, 63), again suggesting this modification to be introduced early. To reconcile these differences, the interdependence of the early modifications m^5^U54 and Ψ55 on m^1^A58 was examined using yeast KOs (60), and the effect of these early modifications on Trm6/Trm61 activity was studied with partially modified tRNAs *in vitro* (64). Presence of m^5^U54 and Ψ55 increases m^1^A58 content in bulk tRNA in the cell, and previous introduction of Ψ55 is necessary for efficient formation of m^1^A58 in *in vitro* transcribed elongator tRNA, but interestingly not initiator tRNA (60, 64). *In vitro*, m^5^U54 additionally stimulates Trm6/Trm61 activity (64). Importantly, as both Trm2 (m^5^U54) and Pus4 (Ψ55) are localized to the nucleus in yeast, it is feasible that these enzymes modify tRNA prior to Trm6/Trm61.

Mass spectrometry has long been used to detect tRNA modifications, and recently nucleic acid isotope labeling coupled mass spectrometry was developed to examine the temporal order of tRNA modification (65). This assay uses stable isotope pulse-chase labeling of HEK293 cultures to observe tRNA modification in newly transcribed tRNA. Using tRNA^Phe^, Ψ55 found to also be very quickly incorporated in human tRNA, followed in this case by m^5^U54, m^1^A58, m^5^C49, and then m^7^G46. In the anticodon stem-loop, m^1^G37 is introduced quickly and slowly converted to yW37, and Cm and Gm are introduced following m^1^G37 (65). Interestingly, in another study, HEK293FT cells lacking Trm6 (and therefore m^1^A58) showed decreased m^5^U/m^5^Um content at position 54 in tRNA^Phe^ and tRNA^Lys^ (66).

Finally, recent advances within the field of tRNA sequencing have made strides toward distinguishing many tRNA modifications (67, 68). In comparison to the experiments described above, next-generation sequencing approaches are advantageous as high-throughput studies, which allow for study of all tRNA species individually without extracting specific isoacceptors or examining only overall trends using bulk tRNA. Unlike Illumina sequencing, Nanopore sequencing can directly sequence RNA without the need for complementary DNA preparation and additionally can distinguish several modifications from canonical nucleotides during this process. Recently, Nano-tRNA-seq recapitulated the requirement of the yeast *pus4* gene (catalyzing Ψ55) for efficient m^5^U54 and m^1^A58 formation (67). As Nanopore sequencing is a single-molecule method that can determine the presence of modifications within individual tRNAs, this technique is likely to uncover more tRNA modification networks, as has recently been accomplished for rRNA (59, 67, 69). In contrast to Nanopore sequencing, Illumina RNA sequencing requires short reads and thus RNA is usually fragmented prior to sequencing. Because of this step, Illumina sequencing is generally regarded to not be a single molecule method, and only the proportion of modifications in a population can be reported. However, since tRNAs are small (76–90 nucleotides), the entire length can be covered by a single Illumina sequencing read, especially due to advances in overcoming tRNA folding and bulky modifications during complementary DNA preparation (70, 71). Thus, a recent computational pipeline termed single-read analysis of crosstalks was developed to treat Illumina tRNA sequencing reads in a “pseudo-single molecular manner” to examine interdependencies between tRNA modification, aminoacylation, and fragmentation (72). Application of this technique to previously published Illumina tRNA sequencing datasets confirmed several modification crosstalks in tRNA, and identified new interdependencies, several of which involve the m^1^A58 modification (72).

The studies described above have identified several interdependencies between the introduction of tRNA modifications *in vivo*, particularly in yeast and humans. While the results above determined the presence of tRNA modification circuits *in vivo*, we are still lacking an understanding of the mechanisms that give rise to several of these interdependences. A few studies have addressed this by using partially modified tRNA and purified enzymes to compare the binding and affinity of these enzymes for unmodified and partially modified tRNA (64, 73, 74, 75). By studying the binding and modification preferences of the bacterial enzymes responsible for m^7^G46, m^5^U54, and Ψ55 (TrmB, TrmA, and TruB, respectively), we uncovered that TruB has a tighter affinity and faster modification rate for unmodified tRNA compared to tRNA that contains one or more additional modifications (74), suggesting the early introduction of Ψ55 is likely to also occur in bacteria. The affinity of TrmA was increased for tRNA containing Ψ55; however, methylation of this Ψ55 tRNA was slower than unmodified tRNA. It is plausible that m^5^U54 acts early, but potentially following Ψ55 introduction in bacteria. *In vivo* studies will need to be accomplished to determine the preferred order of modification in bacteria. Finally, corroborating the introduction of m^7^G somewhere during the middle of tRNA maturation, TrmB did not have a binding or modification preference for tRNA containing or lacking modifications (74), and *in vivo* studies suggest a requirement of m^7^G46 formation for later incorporation of acp^3^U47 in bacterial tRNA (13). Taken together, *in vivo* detection of modifications combined with enzymatic studies are likely to uncover more tRNA modification circuits and their mechanisms in the future.

## Many tRNA modifying enzymes affect tRNA folding and structural dynamics

Although the vast majority of tRNAs adopt the canonical L-shaped tertiary structure in the absence of any modifications, tRNA modifying enzymes have been shown to enhance tRNA folding and structural dynamics through two independent processes: (1) the chemistry of the modified nucleotide altering tRNA structure or dynamics or (2) the act of the tRNA modifying enzyme binding and accessing its target base while locally unfolding the tRNA structure, providing potentially misfolded tRNA a second chance at correctly folding. Each of these topics has been recently reviewed elsewhere (16, 17, 18). Below, we will briefly summarize how tRNA modifying enzymes impact tRNA structure.

Only very few tRNAs require modification in order to adopt the canonical tRNA structure and these instances primarily involve mitochondrial tRNAs, which often feature an atypical secondary structure, sometimes lacking canonical tRNA features such as the D and/or T arms (76). In particular, unmodified human mitochondrial tRNA^Lys^ primarily adopts an extended hairpin conformation secondary structure, but introduction of m^1^A9 shifts the structural equilibrium to the canonical L-shape fold by disrupting a base-pair between A9 and U64 (77). Similarly, unmodified mitochondrial tRNA^Asp^ exists in several conformations *in vitro*, but presence of its native modifications (m^1^A9, m^1^G10, Ψ27, and Q34) stabilizes the canonical cloverleaf structure (78). Also, m^1^A9 has been shown to be important for the overall structure of T arm-less mitochondrial tRNAs from nematodes (79). Finally, m^2,2^G at different positions within the tRNA D arm functions to restrict the folding of tRNAs that can fold into multiple conformations (80, 81).

In contrast to the examples above, the vast majority of tRNAs do not strictly require any modifications to adopt the canonical L-shaped tertiary structure (82). However, various biophysical techniques have demonstrated how modifications subtly affect tRNA structure and thermodynamics (83). For example, several tRNA body modifications collectively contribute to the stability of tRNA^Ser^ as demonstrated by loss of tRNA thermal stability in their absence (75), NMR studies have shown the contribution of m^1^A58 for folding of the tRNA elbow for yeast tRNA_i_^Met^ (64), CD spectrometry has demonstrated Ψ has enhanced base-stacking propensity compared to uridine (84), and molecular dynamics simulations have shown various hypermodifications at position 37 result in different nucleotide glycosidic and backbone conformations compared to unmodified tRNA (85). Thus, modifications play subtle, but important roles to enhance the structure and dynamics of tRNA.

In addition to the role of modifications in augmenting tRNA structure, the enzymes that modify tRNAs themselves influence tRNA folding independently of their modification activity. Following decades-old research showing expression of catalytically inactive TruB rescues the coculture growth defect of *ΔtruB E. coli* (86), TruB was the first modification enzyme proven to be a tRNA chaperone (17). Upon binding tRNA, TruB disrupts tertiary interactions between the D and T arms, thus providing a potentially misfolded tRNA multiple chances at refolding and obtaining its correct fold (17). Subsequently, bacterial TrmA was additionally characterized to be a tRNA chaperone (16). So far, *E. coli* TrmA and TruB have been the only tRNA modifying enzymes categorized as tRNA chaperones; however, likely several more tRNA chaperones are yet to be discovered (18), including TrmA and TruB’s eukaryotic homologs, Trm2 and Pus4. Evidence supporting this possibility includes the observation that the expression of catalytically inactive Trm2 rescues accumulation of tRNA^Ser^ variants (87), and that the bovine mitochondrial Trm2 homolog lacks a key catalytic residue vital for methylation suggesting tRNA binding (and possibly folding) is more conserved than tRNA modification by Trm2 (88). Additional tRNA modifying enzymes that disturb tRNA tertiary interactions and are likely to act as tRNA chaperones include ArcTGT, which remodels tRNA from its typical L-shaped structure into an alternative structure, termed the “lambda (λ)” form (see tRNA bound to ArcTGT, Fig. 3) in order to access its target base (89). Finally, a catalytically inactive variant of the methyltransferase Trm1 can rescue pre-tRNA maturation in *Schizosaccharomyces pombe* and functions redundantly with the tRNA chaperone La, suggesting Trm1 may also act as a tRNA chaperone (90, 91, 92).

## tRNA modifications affect tRNA cellular stability

In general, tRNAs are thought to be very stable within cells, with half-lives similar to that of ribosomal RNA (2). However, tRNAs are subjected to different quality control mechanisms during their lifecycle, wherein defective (pre-)tRNAs are repaired or degraded. These mechanisms have been best studied in *S. cerevisiae*, where two main tRNA decay pathways have been identified: nuclear surveillance and rapid tRNA decay (RTD). In tRNA nuclear surveillance, pre-tRNA_i_^Met^ lacking m^1^A58 or elongator pre-tRNAs with unprocessed 3ʹ ends are bound by the TRAMP complex. TheTrf4 subunit of this complex subsequently polyadenylates the tRNA 3ʹ end, followed by degradation *via* 3ʹ-to-5ʹ exonucleolytic cleavage by Rrp6 as part of the nuclear exosome (93). Whereas the nuclear surveillance pathway primarily monitors pre-tRNAs in the nucleus, the RTD pathway instead acts predominantly on mutation-containing or hypomodified cytoplasmic tRNAs with unstable acceptor/T stems using a different machinery to degrade tRNAs in the 5ʹ-to-3ʹ direction. Here, Met22 and the exonucleases Xrn1 and Rat1 work together to degrade tRNAs with acceptor stems that leave the 5ʹ tRNA termini exposed (94).

Although these decay pathways act on hypomodified tRNAs, the exonucleases involved in these processes are hypothesized to recognize their substrates based on an aberrant or unstable tRNA fold, thus explaining why not all hypomodified tRNAs are degraded and why the RTD pathway is most active in yeast grown at high temperatures (95). Interestingly, overexpression of a single specific tRNA is often sufficient to suppress phenotypes associated with RTD. For example, yeast lacking the methyltransferases *trm8* and *trm4* are slow growing at high temperatures (15). Although both methyltransferases have a wide range of substrate tRNAs in yeast, strikingly, examination of tRNA abundance revealed only tRNA^Val^_(AAC)_ levels are decreased, and the phenotype associated with this strain can be suppressed by overexpressing this tRNA. This trend has also been demonstrated for other tRNA modification combinations, with other examples listed in Table 3. Thus, it may be that not all tRNAs require all modifications; instead, certain modifications may be more important in specific tRNAs than others (96).

**Table 3:** tRNA modifications known to influence the cellular stability of one or more tRNAs

Modification enzyme (modification)	Affected tRNA(s)	Organism, exacerbating conditions, mechanism (if known)	Ref.
m^7^G46 (Trm8/Trm82) and m^5^C48/49 (Trm4)	tRNA^Val^_(AAC)_ and tRNA^Cys^_(GCA)_ to a smaller extent	*S. cerevisiae*, heat stress (37 °C); RTD	(15)
m^7^G46 (Trm8/Trm82) and D47 (Dus3)	tRNA^Val^_(AAC)_ and tRNA^Cys^_(GCA)_ to a smaller extent	*S. cerevisiae,* heat stress (37 °C); RTD	(15)
m^7^G46 (Trm8/Trm82) and Ψ13 (Pus7)	tRNA^Val^_(AAC)_	*S. cerevisiae,* heat stress (37 °C); RTD	(15)
m^7^G46 (Trm8/Trm82)	tRNA^Tyr^_(GUA)_ and tRNA^Pro^_(AGG)_ to a smaller extent	*S. pombe*, heat stress (38 °C); RTD	(201)
m^7^G46 (METTL1/WDR4) and m^5^C48/49 (NSUN2)	tRNA^Val^_(AAC)_	*H. sapiens* (HeLa cell line) exposed to 5-fluorouracil; mechanism unknown	(186)
m^7^G46 (TrmB)	tRNA^Phe^, tRNA^Ile^	*T. thermophilus*, heat stress (80 °C)	(181)
ac^4^C12 (Tan1) and Um44 (Trm44)	tRNA^Ser^_(CGA)_ and tRNA^Ser^_(UGA);_ tRNA^Leu^_(GAG)_ to a smaller extent	*S. cerevisiae*, particularly at high temperatures; exacerbated when grown in glycerol; RTD	(202)
m^2,2^G26 (Trm1) and m^5^C48/49 (Trm4)	tRNA^Ser^_(CGA)_ and tRNA^Ser^_(UGA)_	*S. cerevisiae*, particularly upon heat stress; RTD	(203)
m^2,2^G26 (Trm1)	Several Trm1 substrate tRNAs	*S. pombe*, particularly when *sla1* (La protein) is also deleted	(92)
m^1^A58 (Trm6/Trm61)	pre-tRNA_i_^Met^	*S. cerevisiae* (RTD and nuclear surveillance) and *S. pombe* (RTD), particularly in heat stress	(61, 62, 63)
m^7^G46 (METTL1/WDR4)	Several METTL1 substrate tRNAs	*H. sapiens* (glioblastoma multiforme cell line LNZ308)	(100)
s^4^U8 (ThiI)	Several ThiI substrate tRNAs	*Vibrio cholerae* during stationary phase; bacterial RNA degradosome (RNase E)	(97)
s^4^U8 (ThiI) and m^5^U54 (TrmA)	tRNA^Tyr^	*V*. *cholerae* during stationary phase; bacterial RNA degradosome (RNase E)	(97)
s^4^U8 (ThiI) and Ψ55 (TruB)	tRNA^Tyr^	*V*. *cholerae* during stationary phase; bacterial RNA degradosome (RNase E)	(97)
m^1^G9 (Trm10)	tRNA^Trp^	*S. cerevisiae*, 5-fluorouracil; unknown (mediated by Met22, but Xrn1 and Rat1 nucleases are not involved)	(204)
m^5^C38 (Dnmt2) and m^5^C34 (NSun2)	tRNA^Asp^_(GTC)_ and tRNA^Gly^_(GCC)_	*M*. *musculus* embryonic stem cells (MEFs); mechanism unknown	(205)

In contrast to yeast, the mechanisms of tRNA decay in other organisms including bacteria and mammals are less studied. Work in *Vibrio cholerae* has demonstrated that several different tRNAs lacking elbow modifications including s^4^U8, m^5^U54, and Ψ55 are subject to decay by the RNA degradosome, which is comprised of the helicase RhlB, enolase, the polynucleotide phosphorylase PNPase, and RNase E (97). Furthermore, KO or knockdown of METTL1 and/or WDR4 in different human cells lines results in decreased abundances of many tRNAs that normally contain m^7^G by an unknown decay mechanism (98, 99, 100). Overexpression of METTL1 conversely increases the cellular abundance of several m^7^G-containing tRNAs, which has been implicated in driving several types of cancer through altering translation in a codon-biased manner (98, 99, 100). These examples of bacterial and human tRNA degradation appear to affect tRNAs more broadly than the yeast instances described earlier wherein only a small number of specific tRNAs are targeted in the absence of modification enzymes (Table 3).

## tRNA modifications during canonical tRNA function: protein translation

The canonical role of tRNAs is to bring amino acids to the ribosome for protein synthesis, which we will briefly discuss here. First, tRNAs are aminoacylated by their cognate aminoacyl-tRNA (aa-tRNA) synthetase (aaRS) to form aa-tRNA. Subsequent steps depend on the identity of the tRNA, but as the majority of tRNAs are elongator tRNAs, we focus here on elongator tRNAs and the specific case of initiator tRNA is addressed in a dedicated section. Bacterial elongation factor Tu (EF-Tu, eEF1A in eukaryotes) in its GTP state binds an aa-tRNA to form the EF-Tu•aa-tRNA•GTP ternary complex, which delivers aa-tRNA to the ribosomal acceptor (A) site. Following peptide bond formation between the peptidyl-tRNA in the peptidyl (P) site and the aa-tRNA, bacterial elongation factor G (EF-G, eEF2 in eukaryotes) catalyzes translocation, wherein the uncharged tRNA moves to the exit (E) site and peptidyl-tRNA moves to the P site. When the ribosome encounters a stop codon (UAG, UGA, or UAA), bacterial release factors 1 or 2 (RF1 or RF2) promote hydrolysis of the peptidyl-tRNA, and release factor 3 help RF1 or RF2 dissociate from the ribosome.

Unsurprisingly, tRNA modifications within the anticodon or adjacent to the anticodon within the tRNA anticodon stem loop have been shown to play a variety of direct and sometimes essential roles during translation, which will be discussed below. However, tRNA modifications within the tRNA elbow can also impact this process. Here, we summarize known roles for tRNA modifications during tRNA aminoacylation, elongation/initiation factor binding, translation initiation, and translation elongation. Presumably, tRNA modifications do not play a role in translation termination as a protein-based process.

## tRNA modifications affect tRNA aminoacylation

To maintain the fidelity of protein synthesis, it is paramount that aminoacyl-tRNA synthetases charge only their cognate tRNA and not other similar tRNA species. Although seemingly not a common theme for tRNA aminoacylation, there are cases where tRNA modifications act as determinants or antideterminants for aaRS, which has been recently reviewed in (101). In particular, it is well-known that lysidine (L) 34 determines the aminoacylation and codon identity of tRNA^Ile2^ as an isoleucyl-rather than methionyl-tRNA (102). Moreover, *in vitro* aminoacylation of unmodified tRNAs often tends to be inefficient (103), suggesting a role for modifications in fine-tuning the affinity of tRNA for its cognate synthetase or enhancing the esterification reaction. Finally, as discussed above, proper tRNA folding is a prerequisite for tRNA charging; thus, the tRNA chaperone activity of certain modification enzymes is likely to increase the proportion of aminoacylated tRNAs in cells (16, 17, 18, 104) which we recently confirmed for *E. coli* TrmA and TruB (105). Interestingly, the aaRS SerRS displays crosstalk with the tRNA methylating enzyme Trm140 (yeast) and its homolog METTL6 (humans), whereby SerRS immunoprecipitates Trm140/METTL6 in a tRNA-dependent manner, and SerRS presence increases the tRNA^Ser^ methylase activity of these enzymes both *in vitro* and *in vivo* (106, 107, 108, 109), suggesting a codependence of these processes.

In Table 4, we have summarized tRNA modifications that are known to affect the activity of certain aaRS. For the majority of cases, these effects have been determined by comparing the steady-state kinetic parameters for aaRS with native (fully modified), unmodified, or partially modified tRNAs *in vitro*.

**Table 4:** tRNA modifications affecting aminoacylation

Modification (enzyme)	Affected aaRS	Affected tRNA	Organism	Evidence/mechanism	Ref.
mnm^5^s^2^U34 (MnmE, MnmG; MnmA)	GluRS	tRNA^Glu^	*E. coli*	*In vitro* aminoacylation kinetics	(206, 207, 208, 209, 210)
	LysRS	tRNA^Lys^			
	GlnRS	tRNA^Gln^			
L34 (aka k^2^C, TilS)	IleRS	tRNA^Ile^_CAU_	*E. coli*	tRNA^Ile^_CAU_ lacking L34 is efficiently aminoacylated by MetRS and not IleRS. L34 modification serves as a positive determinant for IleRS and a negative determinant for MetRS	(102)
	MetRS	tRNA^Ile^_CAU_	*E. coli*		
I34 (Tad2/3)	IleRS	tRNA^Ile^_IAU_	*S. cerevisiae*	Introduction of only I34 increases the *k*_cat_ of aminoacylation 12-fold compared to an unmodified transcript	(103)
t^6^A37 (TsaBDE)	IleRS	tRNA^Ile^_GAU_	*E. coli*	Faster aminoacylation (∼25-fold) of tRNA^Ile^_GAU_*in vitro* by *E. coli* IleRS when t^6^A37 is present	(191)
agm^2^C34 (TiaS)	IleRS	tRNA^Ile^_agm2CAU_	archaea (*Haloarcula marismortui*)	Analog of lysidine; similarly is responsible for Ile identity	(211, 212)
m^1^G37 (TrmD)	ProRS	tRNA^Pro^_CGG_	*E. coli*	17-fold reduction in catalytic efficiency for tRNA^Pro^_CGG_ aminoacylation by ProRS	(213)
m^1^G37 (Trm5)	ArgRS	tRNA^Asp^	*S. cerevisiae*	methylation prevents misacylation of tRNA^Asp^ by ArgRS by reducing v_max_ and increasing *K*_M_	(214)
Ψ35 (Pus1)	TyrRS	tRNA^Tyr^	*S. cerevisiae*	*In vitro* aminoacylation kinetics	(215)
Ψ34/Ψ36 (Pus1)	IleRS	tRNA^Ile^_ΨAΨ_	*S. cerevisiae*	40-fold decrease in catalytic efficiency of transcript compared to native tRNA (additional native tRNA modifications may also be involved)	(103)
yW37 (Trm7)	PheRS	tRNA^Phe^	*S. cerevisiae* and *S. pombe*	Decreased cellular aminoacylation for *Δtrm7* cells compared to wildtype when grown in minimal media	(72, 216)
m^1^A9 (unknown)	PheRs	mt-tRNA^Phe^	*Ascaris suum*	Decreased *in vitro* aminoacylation for at least these two T-armless tRNAs	(79)
	MetRS	mt-tRNA^Met^			
m^2,2^G26 (Trm1)	SerRS	tRNA^Ser^_(CGA/UGA)_	*S. pomble*	∼25% decrease for *in vivo* aminoacylation levels	(92)
Ψ31 (Pus6)	MetRS	tRNA_e_^Met^	*S. cerevisiae*	Pus6 presence increases amount of tRNA^Met^ immunoprecipitated by MetRS	(217)

## Translation initiation: t^6^A37 restricts codon reading by initiator tRNA

Unlike EF-Tu/eEF1A, which binds all elongator aa-tRNAs, initiation factor 2 (IF2, bacteria; eIF2, eukaryotes) is specific to binding only initiator tRNAs (fMet-tRNA^fMet^, bacteria; Met-tRNA_i_^Met^, eukaryotes). *In vitro* transcribed mammalian tRNA_i_^Met^ has the ability to be aminoacylated and form a complex with eIF2•GTP, but a comparison to the interactions with fully modified tRNA_i_^Met^ has yet to be conducted (110). Although modifications to tRNA_i_^Met^ do not appear to affect binding to eIF2, in plants and fungi, presence of a 2′-O-phosphoribosyl modification of the tRNA_i_^Met^ ribose at position 64 formed by Rit1 acts as a steric block preventing initiator tRNA from binding to eEF1A (111, 112). Absence of this modification allows Met-tRNA_i_^Met^ to bind eEF1 *in vivo* (113). In contrast, the sequence of vertebrate tRNA_i_^Met^ itself acts to prevent elongation factor binding in a modification-independent manner (114). In bacteria, IF2 first binds the ribosome to then recruit fMet-tRNA^fMet^. To the best of our knowledge, all studies of bacterial translation initiation *in vitro* have used native fMet-tRNA^fMet^ (115), and no studies examining the role of modifications for bacterial initiation have been conducted.

Mammalian unmodified tRNA_i_^Met^ can effectively form 43S preinitiation and 48S initiation complexes *in vitro*, and fully assembled 80S ribosomes produce methionyl-puromycin at similar speeds *in vitro* with unmodified and modified initiator tRNAs (110). Thus, modifications to tRNA_i_^Met^ do not seem to be essential for translation initiation; however, at least one tRNA modification works to fine-tune this step. Several lines of *in vivo* evidence suggest the highly conserved t^6^A37 modification is important for restricting translation initiation to AUG codons in eukaryotes (116, 117, 118). Interestingly, t^6^A37 is found in eukaryotic initiator tRNAs, but is noticeably absent in bacterial tRNA^fMet^, even though this modification is present in nearly all other tRNAs with an ANN anticodon in bacteria (N = any nucleotide). Unlike eukaryotic tRNA_i_^Met^, which rarely decodes non-AUG codons, bacterial tRNA^fMet^ effectively decodes GUG and UGG codons, supporting the role of t^6^A37 of preventing initiation at non-AUG codons in eukaryotes (117).

## Several roles for tRNA modifications during translation elongation

Before delivery to the ribosome, elongator tRNAs must form a ternary complex with EF-Tu/eEF1A. Since EF-Tu/eEF1A must bind virtually all aa-tRNAs regardless of sequence, it is unsurprising that mutations in the tRNA sequence, that do not perturb tRNA tertiary structure, do not greatly affect Phe-tRNA^Phe^ affinity for EF-Tu•GTP (119). In a similar manner, Phe-tRNA^Phe^ lacking modifications binds EF-Tu•GTP with only an approximately 1.5-fold lower affinity compared to fully modified Phe-tRNA^Phe^ (120). Likewise, affinities of unmodified Gly-tRNA^Gly^, Val-tRNA^Val^, Ala-tRNA^Ala^, and Gln-tRNA^Gln^ binding EF-Tu are no more than three-fold lower compared to their modified counterparts (121, 122, 123). Presumably, EF-Tu•GTP displays a similar lack of preference between other modified and unmodified aa-tRNA species, and eEF1A probably behaves in a similar manner to EF-Tu. As EF-Tu only contacts the tRNA acceptor stem and T arm (Fig. 3), which do not harbor many modifications, the small increases in affinity observed for elongation factors in binding modified tRNAs can likely be attributed to tRNA modification enzymes fine-tuning the L-shaped tRNA tertiary structure and tRNA dynamics. Interestingly, unusual T-armless tRNAs in nematode mitochondria require m^1^A9 for effective binding of EF-Tu (79). For the special case of selenocysteine translation at UGA codons, the selenocysteine tRNA-specific elongation factor (SelB) is specific for binding only Sec-tRNA^Sec^ (124). The affinities of *apo* SelB for modified and unmodified Sec-tRNA^Sec^ are almost identical, suggesting SelB does not require modifications for tRNA binding (125).

**Figure 4:**
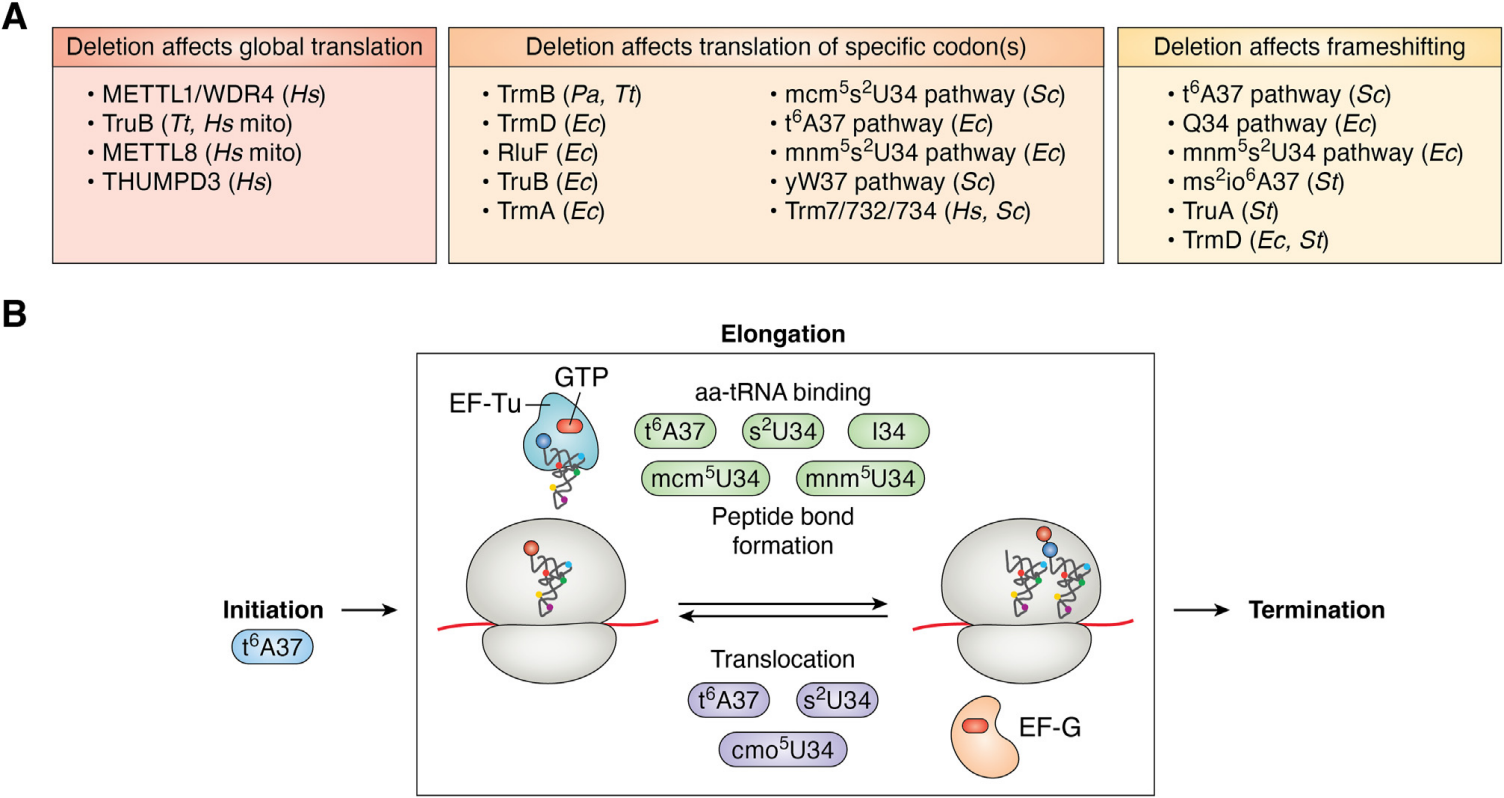
**Summary of the roles of modifications during different steps of translation.***A*, tRNA modifying enzymes whose absence *in vivo* affects global translation, specific codon translation, or frameshifting. Model organism used in each study is indicated in parenthesis. *Hs: Homo sapiens*, *Tt: Thermus thermophilus*, *Pa: Pseudomonas aeruginosa*, *Ec: Escherichia coli*, *Sc: Saccharomyces cerevisiae*, *St: Salmonella typhimurium*, mito: mitochondria. *B*, general schematic of the overall steps of translation. Modifications found to play roles within each step are highlighted within colored boxes.

Once part of a ternary complex, tRNAs bind to the ribosome in a complex, multistep process. In brief, a translating ribosome reversibly samples aa-tRNA•EF-Tu•GTP ternary complexes, forming initial binding complexes. If the ribosome next identifies a cognate codon:anticodon pair, GTPase activation and subsequent GTP hydrolysis by EF-Tu is triggered, followed by the release of inorganic phosphate. Dissociation of EF-Tu may proceed or follow accommodation of aa-tRNA in the ribosomal A site, which is then followed either by proofreading, wherein aa-tRNA may be ejected from the ribosome, or rapid peptide bond formation (126, 127). The next step of elongation is translocation, which prepares the ribosome for another round of elongation (Fig. 4).

tRNA modifications have been shown to play a multitude of roles during translation elongation, including aiding tRNA binding to the ribosome, enhancing decoding of mRNA codons, restricting decoding to only cognate codons, preventing proofreading at cognate codons, aiding in translocation, and enhancing or suppressing various forms of ribosomal frameshifting, and several of these processes have been reviewed extensively elsewhere (9, 10, 128). In the paragraphs below, we will detail general roles for tRNA modifications in translation and the approaches used to study these. Roles for several specific modifications are summarized in Table 5.

**Table 5:** Examples of tRNA modifications influencing translation elongation

Modification (enzymes)	Codons and/or tRNAs affected; model organism	Experiments	Ref.
mcm^5^s^2^U34 (ELP and URM1 pathway)	AAA, GAA, and CAA; *S. cerevisiae*	Proteome analysis, tandem-codon translation reporter assays, *in vitro* translation assays	(218)
s^2^U moiety of mcm^5^s^2^U34 (URM1 pathway)	tRNA^Lys^_UUU_ reading AAA codon; *S. cerevisiae*	*In vitro* translation kinetics	(219)
mcm^5^(s^2^)U34 (Trm9)	AGA, AGG, and GAA codons; *S. cerevisiae*	Tandem-codon translation reporter assays, proteomics	(134, 220)
Ψ35 (RluF)	tRNA^Tyr^_GUA_; *E*. *coli*	Tandem-codon translation reporter assays	(221)
m^7^G46 (TrmB)	tRNA^Phe^_GAA_ reading UUU and UUC, tRNA^Asp^_GUC_ reading GAC and GAU codons; *P. aeruginosa*	Tandem-codon translation reporter assays	(178)
m^7^G46 (METTL1)	Global translation in human HuCCT1 and RBE cell lines, particularly for codons read by m^7^G modified tRNAs	Polysome profiling, ribosome profiling, and pulse-labeling	(98)
mnm^5^U34 (MnmE/G), m^1^G37 (TrmD), t^6^A37 (TsaBCDE)	*E. coli* tRNA^Glu^_UUC_, tRNA^Pro^_UUC_; both tRNA^Ile^_GAU_; and tRNA^Asn^_GUU_, respectively	*In vitro* translation (PURExpress system)	(222)
Cm32 and Gm34 (Trm7)	Human, mouse and yeast global translation, Phe UUU and UUC codons particularly affected	Polysome profiling, tRNA overexpression, ribosome profiling	(195, 197, 223)
yW37 (Tyw2-4)	tRNA^Phe^_GAA_ reading UUU	*In vitro* translation kinetics	(141)
t^6^A37 and mnm^5^U34 (TdcBCDE, MnmEG)	tRNA^Lys^ anticodon stem loop; *E. coli*	*In vitro* ribosomal A site binding and translocation	(224)
cmo^5^U34 (CmoA, CmoB)	tRNA^Val1^ anticodon stem loop reading GUU; *E. coli*	*In vitro* ribosomal A site binding and translocation	(224)
Ψ55 (TRUB1)	Global mitochondrial translation, *H. sapiens* (HeLa cell line)	Comparison of specific protein abundance by Western blot	(225)
m^7^G (TrmB)	Global translation, *T*. *thermophilus* under heat stress	^35^S-pulse labeling	(181)
Ψ55 (TruB)	Global translation, *T*. *thermophilus* under cold stress	^35^S-pulse labeling	(175)
m^5^C34, 48 (Trm4)	TTG codons; *S. cerevisiae*	Tandem-codon translation reporter assays, proteomics	(226)
m^3^C32 (METTL8)	Global translation, *H. sapiens* HCT116 cell line; mitochondrial translation, particularly at codons read by tRNA^Tyr^ and tRNA^Ser^, HAP1 human cells	Polysome profiling shows subtle affects; ribosome profiling displays stalls at A and/or P site	(107)
m^3^C32 (METTL6)	Translation efficiency of several genes, mouse ESCs	Ribosome profiling	(227, 228)
m^5^C38 (Dnmt2) and m^5^C34 (NSun2)	Global translation in MEFs and mouse embryos	^35^S-pulse labeling and polysome profiling	(205)
m^2^G6 (THUMPD3)	Global translation in human HEK293T cells	Polysome profiling	(229)
Q34 (TGT)	Global translation, especially at Q34-decoded codons; HeLa cells	Polysome profiling, ribosome profiling	(142)
m^1^G37 (TrmD)	Prevents tRNA^Pro^ decoding CC(C/U)-C/U slippery sequences from frameshifting; additionally prevents ribosomal stalling at select codons; *E. coli*	Structural studies, reporter assays, *in vitro* ribosome binding and translation studies, ribosome profiling	(230, 231, 232, 233, 234)
I34 (ADAT2/3)	Altered translation for a subset of mRNAs	Ribosome profiling, polysome profiling	(235, 236)

Of course, all maturation processes affected by tRNA modifications can influence translation (*e.g.* modifications that lead to lower concentrations of cellular tRNA or decreased aminoacylation levels), but modifications themselves additionally play roles in protein synthesis. Traditional *in vivo* experiments to examine the role of tRNA modifications on translation generally compare a tRNA modifying enzyme KO to its corresponding WT strain, (*e.g.* pulse-labeling, translation reporter experiments, and examination of single protein expression by Western blotting) and are often unable to distinguish between a direct role of a modification in translation and the effects of the modification for a previous step of tRNA maturation. In contrast, *in vitro* assays using purified ribosomes and translation components and differently modified tRNA can often demonstrate direct roles for tRNA modifications in translation and narrow down the affected step in translation. As many tRNA modification enzymes modify only select tRNAs and the codon content of mRNAs often plays a role in fine-tuning translation efficiency, it is important to examine how codons are affected differently by the loss of a tRNA modification enzyme. High-throughput experiments to examine translation such as ribosome profiling (129, 130) can distinguish mRNA codons (and thus their corresponding tRNAs) that are most affected by modification enzyme deletion and provide clues into how translation is altered by examining whether ribosome pausing occurs when the codon is in the A site or P site. For example, ribosome profiling has been systematically accomplished for 57 yeast deletion strains lacking genes involved in tRNA modification (131). Similarly, ribosome-bound tRNA capture (132) can be used to identify which tRNAs are bound to ribosomes, whether they are in the A or P site, which modifications ribosome-bound tRNAs contain, and how this compares to the overall cellular tRNA population. Further, by comparing the proteome between a tRNA modifying enzyme KO and its corresponding WT, codon-bias can be examined for upregulated and downregulated proteins to identify codons that are potentially affected by tRNA modification (105, 133, 134).

Since non-Watson-Crick base-pairs are tolerated at the “wobble” position between the third nucleotide of a codon and the first nucleotide (N34) of a tRNA anticodon, modifications at the wobble position in tRNAs are frequently found to restrict, enhance, or rarely, change decoding. In particular, uridines at position 34 are modified at a high frequency and have been extensively studied and reviewed in (10, 135, 136). Likewise, the deamination of adenosine forming the edited based inosine (I) is common at the wobble position. With the ability to base pair with A, C, and U bases, I34 expands base pairing capacity and is essential for the decoding of certain codons in bacteria and eukaryotes (137, 138, 139, 140). Modifications adjacent to the anticodon, especially at the conserved purine 37, often play roles in stabilizing the codon:anticodon base-pairing interaction by enhancing base-stacking (141). By optimizing the speed of translation, anticodon arm modifications can contribute to protein folding, leading to accumulation of aggregated proteins in the absence modifications including Q34, mcm^5^U34, and t^6^A37 (10, 117, 133, 142). Although the impact of many anticodon stem-loop modifications on translation have been studied, it remains unknown whether tRNA modifying enzymes that target nucleotides outside of the tRNA anticodon (*i.e.* in the tRNA elbow) directly contribute to translation. By enhancing the structure, folding, and dynamics of the tRNA elbow, these modifications may promote tRNA movement through the ribosome. Supporting this notion, several tRNA elbow modifications have been indirectly implicated in protein synthesis (Table 5).

## Alternative roles of tRNAs and tRNA modifying enzymes

tRNAs across all domains of life participate in roles outside of protein synthesis. Noncanonical roles for aminoacylated tRNAs include the delivery of amino acids for biosynthesis of amino acids (143), antibiotics (144), tetrapyrroles (145), and various components of the cell wall (146). Additionally, aa-tRNAs deliver destabilizing amino acids for N-terminal proteolytic tagging by the N-end rule pathway to regulate the half-life of proteins in cells (147). Uncharged tRNAs regulate gene expression by acting as nutrient sensors, for example in the bacterial stringent response and in amino acid starvation conditions in eukaryotes (148). Moreover, tRNAs inhibit intrinsic apoptosis by binding cytochrome c (149). To the best of our knowledge, tRNA modifications have not been shown to play direct roles in the processes described above to date. Conversely, roles for tRNA modifications in virus biology have been defined. For example, retroviruses, including HIV, utilize a tRNA containing the m^1^A58 modification formed by TRMT6/TRMT61 as a primer for viral reverse transcription (150). Using HIV-1 as a model, it has been shown m^1^A58 is necessary to generate the so-called “plus-strand strong-stop” (151) during reverse transcription, which is required for proper plus strand synthesis and viral genome integration (66, 152, 153, 154, 155). Further supporting the role of m^1^A58 by TRMT6/TRMT61 in HIV replication, TRMT6 is required for efficient accumulation of viral proteins (66). Additional tRNA modifications have been implicated in viral pathogenesis by altering translation in the host (156, 157, 158, 159).

In addition to noncanonical functions for full-length mature aminoacylated and nonaminoacylated tRNAs described above, the specific fragmentation of tRNAs gives rise to tRNA-derived small RNAs (tDRs, also known by other names including tRNA fragments) (160, 161). tDRs are formed by several ribonucleases including angiogenin, and are often upregulated in cellular stress conditions (162). An assortment of tDRs of different types and lengths play a variety of functions in each domain of life in various cellular processes including ribosome biogenesis and gene silencing by binding to complementary nucleic acids and to various proteins (163, 164, 165). Several tRNA modification enzymes have been shown to regulate the biogenesis of tDRs; these cases have been extensively reviewed in (161, 162) and will only be briefly summarized here. In most or all cases identified to date, the presence of a tRNA modification and its corresponding enzyme limits tDR production (162), suggesting tRNA modifying enzymes impart a protective effect against nucleases. For example, loss of m^1^9A by TRMT10A increases formation of 5ʹ-tRNA^Gln^ fragments in TRMT10A-deficient patient lymphoblasts (166) and 2′O-methylation of C34 by the methyltransferase fibrillarin as part of a C/D box small nucleolar ribonucleoprotein complex prevents tRNA^Met^_(CAT)_ cleavage by angiogenin in HAP1 cells (167). The mechanism(s) by which tRNA modifying enzymes control tDR biosynthesis remain unknown but could either be due to binding of the tRNA modifying enzyme regulating the accessibility of tRNA for nucleases or by tRNA modification or folding status acting as determinants for tDR-forming nucleases.

Finally, some tRNA modifying enzymes may have moonlighting activities in other cellular functions. Most interestingly, at least two tRNA modifying enzymes, Mod5 and Pus4, can exist in a prion form in yeast as [*MOD5*^*+*^] and [*BIG*^*+*^] (Better In Growth), respectively (168, 169). [*MOD5*^*+*^] cells have decreased i^6^A37 modification compared to nonprion state WT cells, but [*BIG*^*+*^] cells seem to have similar Ψ55 levels as their counterparts although an increase in translation elongation factor *TEF1/TEF2* mRNA pseudouridylation is observed (168, 169). Interestingly, aggregation of Mod5 protein *in vitro* is stimulated by tRNA binding (170). The Mod5 human homolog TRIT1 additionally forms amyloid fibers *in vitro* and *in vivo* (171). The implications on tRNA modifying enzyme prion formation and tRNA modification has yet to be described.

## Conclusions and perspectives

In summary, we have described how tRNA modifying enzymes affect tRNAs at all stages of the tRNA lifecycle by modifying and folding tRNAs. Together, the research summarized here suggests that deletion of a single tRNA modification enzyme often affects more than the loss of the given modification; instead, the entire tRNA modification status can be altered and the tRNA folding and structural dynamics may be affected. Some hypomodified and potentially misfolded tRNA may be targeted for degradation, affecting cellular tRNA abundance. Alternatively, hypomodified tRNA may be improperly recognized by its interacting factors, which may alter tRNA processing, aminoacylation, translation and/or other noncanonical tRNA functions.

Therefore, comprehensive awareness of the molecular functions of tRNA modifying enzymes is vital to further our understanding of the roles of tRNA modifying enzymes in a variety of organisms, in genetic diseases and cancer (6). Knowing the underlying mechanisms of diseases and whether they are a result of changes in tRNA levels, aminoacylation or other tRNA functions provides clues to developing targeted therapies, *e.g.* by aiming to increase tRNA concentrations or the concentrations of factors interacting with tRNAs. Furthermore, knowledge of the consequences of including or excluding modifications in tRNA is crucial for potential therapeutic development and optimization of tRNA molecules as future medicines for diseases (172, 173). We anticipate that the engineering of tRNA modifications will play a critical role in designing efficient suppressor tRNAs for read-through of premature stop codons or in creating tRNAs delivering noncanonical amino acids to the ribosome. In brief, our understanding of the complex functions of tRNA modifying enzymes for the tRNA lifecycle has sufficiently advanced to enable exciting new opportunities in developing new therapeutics and biotechnological applications.

## Conflict of interest

The authors declare that they have no conflicts of interest with the contents of this article.
